# Computer vision and statistical insights into cycling near miss dynamics

**DOI:** 10.1038/s41598-024-70733-8

**Published:** 2024-09-10

**Authors:** Mohamed Ibrahim

**Affiliations:** 1 Institute of Spatial Data Science, University of Leeds, Woodhouse, Leeds, LS2 9JT UK; 2 Leeds Institute for Data Analytics (LIDA), University of Leeds, Woodhouse, Leeds, LS2 9JT UK

**Keywords:** Computer vision, Deep learning, Cycling near misses, Granger causality, Environmental social sciences, Sustainability, Computational science, Computer science

## Abstract

Across the globe, many transport bodies are advocating for increased cycling due to its health and environmental benefits. Yet, the real and perceived dangers of urban cycling remain obstacles. While serious injuries and fatalities in cycling are infrequent, “near misses”-events where a person on a bike is forced to avoid a potential crash or is unsettled by a close vehicle-are more prevalent. To understand these occurrences, researchers have turned to naturalistic studies, attaching various sensors like video cameras to bikes or cyclists. This sensor data holds the potential to unravel the risks cyclists face. Still, the sheer amount of video data often demands manual processing, limiting the scope of such studies. In this paper, we unveil a cutting-edge computer vision framework tailored for automated near-miss video analysis and for detecting various associated risk factors. Additionally, the framework can understand the statistical significance of various risk factors, providing a comprehensive understanding of the issues faced by cyclists. We shed light on the pronounced effects of factors like glare, vehicle and pedestrian presence, examining their roles in near misses through Granger causality with varied time lags. This framework enables the automated detection of multiple factors and understanding their significant weight, thus enhancing the efficiency and scope of naturalistic cycling studies. As future work, this research opens the possibility of integrating this AI framework into edge sensors through embedded AI, enabling real-time analysis.

## Introduction

Unsafe mobility interactions, either with other road users or infrastructures, are key contributors to unsafe behaviours in cities^1,2^. There has been a surge in the popularity of cycling, both in Europe and globally^3^. The potential health benefits of cycling and its role in reducing environmental pollution have driven planners and policymakers to invest in bike infrastructure, be it for daily commutes or leisure activities^4–7^. To boost cycling, a variety of policies, initiatives, and both tangible and intangible interventions have been introduced across the world^6,8^. In the UK, for instance, Transport for London (TfL) has backed numerous cycling projects, such as cycling superhighways, quiet routes, mini-Hollands, and bike rental schemes to ensure a safer environment for cyclists^9^. However, even with the notable health benefits outweighing the risks of cycling^4^, the risk for people on bikes remains high^10^.

Near miss experiences can shape the perception of cycling as a dangerous activity^1,2, 11, 12^. In the UK, cyclists are estimated to have at least one close pass every six miles they commute^13^. Concerns about collisions or falls deter many from embracing cycling as a means of transportation^1,14, 15^. A UK study involving 244 cyclists and non-cyclists found that a perceived lack of safety was a primary barrier for many against cycling^16^. The apprehension around cycling risks and past close calls have been identified as significant obstacles to wider cycling adoption^17^. These near misses can be attributed to various traffic-related anxieties, including distracted drivers, vehicles passing too closely, door-related incidents, speeding vehicles, aggressive drivers, or being abruptly cut off by a turning vehicle^17^.

Cycling near misses are transport-related, but the factors that contribute to them may or may not be connected to transport^1,14, 18–20^, therefore it’s important to look at the bigger picture. These factors can be related to aspects such as visibility, physical conditions of the built-up areas, interaction among different road users, or behavioural and psychological factors related to the cyclist, in which there is a clear knowledge gap in extracting these factors automatically^2^. When taken as a whole, cycling near misses can be viewed as a type of urban system that evolves in cities as a result of various circumstances and events that might or might not be directly tied to transportation. For interpreting cycling near misses, it is essential to have a solid understanding of the many urban systems that exist in cities as well as their dynamics. Understanding cities and subsequently human mobility through computer vision has shown substantial progress in the last few years^21^.

Artificial Intelligence (AI), specifically, deep learning has significantly advanced our understanding of urban mobility and the dynamics of city life, offering powerful tools to analyse and predict patterns within complex urban environments^2,21^. By utilising large datasets, deep learning models can uncover complex relationships and behaviours in domains such as traffic flow, pedestrian movement, and accident and near accident experiences. A key aspect of applying deep learning to urban mobility involves feature engineering from video streams, such as using optical flow. Optical flow techniques analyse the motion of objects between consecutive frames in video streams, enabling the extraction of meaningful data regarding speed, direction, and density of movement. This approach has been utilised in understanding cycling near misses^22^. In this research, we extend the utility of artificial intelligence in inferring and analysing human risks by introducing a first-hand computer vision and statistical analysis framework that is able to assist city planners and policymakers to detect and analyse cycling near misses and their risk factors. Based on machine intelligence, the tool will automatically analyse cycling near misses from video streams by understanding the interaction between people, the built environment, and the different transport modes. The research will have several benefits in terms of improving road safety. This knowledge of risk factors will enable: (a) individuals (cyclists and other road users) to change their behaviour to minimise risk, (b) transport authorities to plan safer infrastructure and run informed awareness campaigns, and (c) the production of more accurate risk maps, showing which routes are safest for cycling, and what types of incidents to be wary of.

The relevance of this study is substantial in the evolving field of urban cycling safety. By integrating advanced computer vision and statistical analysis into a single framework, this research significantly advances existing methodologies that rely heavily on manual data processing and interpretation. It goes beyond a single AI model. Moreover, traditional approaches to studying cycling near misses are labour-intensive and often suffer from limited scalability due to the sheer volume of video data generated. This new method not only automates the detection and analysis of near misses from video streams but also introduces the ability to systematically identify and quantify risk factors in real-time.

**Fig. 1:**
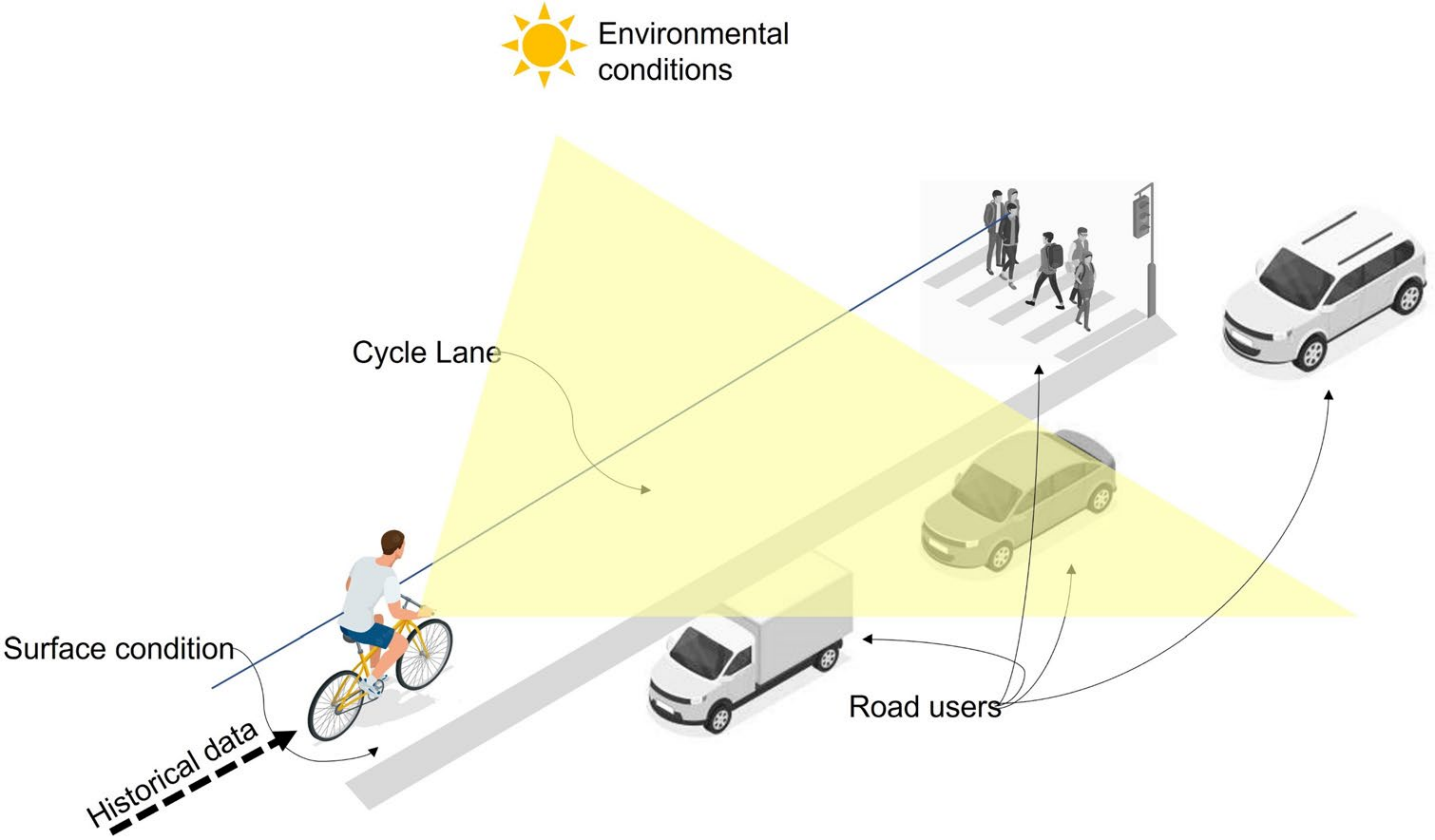
The scope of the presented method for analysing video streams seen from a person on a bike perspective. The scope of the study includes historical data on several risk factors such as environmental conditions (weather, glare), road users detections (cars, humans, bikes, trucks, motorbikes, and buses), and built environment conditions (surface conditions and the presence of a bike lane). This figure is drawn by the Author, Mohamed R Ibrahim.

## Results

### Integrated framework

There is no doubt that advances in computer science in general, or geo-computational methods have led to several advances in geography and understanding urban systems. Seeing cities from the street view adds more dimensions of information and complexity^21^. Capturing these rapid urban changes in day-to-day life through images offers more opportunities to tackle urban dynamics towards a better understanding of cities. We introduced different methods to understand critical events such as cycling near misses and their risk factors. Figure 1 shows the overall scope of our introduced vision framework. This framework can be utilised as a base for generating urban data for multi-purpose urban and transport-related studies. The framework can capture information related to environmental, visual, and built environment conditions coupled with the spatiotemporal context. The framework operates in real-time, achieving 26 frames per second on a single RTX 2080 GPU. The innovation can be seen in detecting critical events and understanding their causes. Also, by applying the same algorithms to active cameras in cities, the model can enable real-time capturing of data. Last, the same methodology can be applied to tackle and classify different urban issues from urban scenes. Coupled with remote sensing image classification methods, the proposed framework can reveal deeper insights into the dynamics of cities. Furthermore, the integration of embedded AI with edge sensors facilitates real-time analytics and data extraction, enabling prompt responses to ongoing events and conditions in urban settings. Beyond cycling near-miss events, this framework can be adapted to detect and analyse various urban phenomena, such as traffic congestion, pedestrian safety, and environmental monitoring.

### Framework stringency index (SI)

Even though each model presented in this research is validated individually, we introduced a Framework Stringency Index (SI) to further evaluate the performance of the individual models for the given task of analysing cycling near misses. What makes this index unique is that it does not only include the performance of each model, but it also takes into account its importance in understanding cycling near misses in terms of the weighting of the variables it generates in the regression models.

**Table 1:** The summary of the results of the introduced framework.

Models	Factors	AP^a^	Statistical weight^b^	Norm. weight^c^
Model1-NightNet	Nightime	0.885	0.268	0.101
	Daytime	0.885	0.120	0.045
	Dawn_dusk	0.885	0.417	0.157
Model2-GlareNet	Glare	0.883	0.099	0.037
Model3-PrecipitationNet(b)	Clear	0.959	0.117	0.044
	Rain	0.959	0.281	0.106
	Snow	0.959	0.199	0.075
Model4-FogNet	Fog	0.862	0.241	0.091
Model5-SlipNet	Wet surface	0.918	0.241	0.091
Model6-Object_detection	Person	0.75	0.074	0.028
	Bicycle	0.79	0.201	0.076
	Car	0.81	0.089	0.034
	Bus	0.77	0.100	0.038
	Motorbike	0.81	0.368	0.139
	Truck	0.77	0.034	0.013
Model7-Cyclinglane	Cyclinglane	0.91	0.1235	0.047

^a^The average precision calculated for each model on the test sets.

^b^The absolute value of the statistical weight of the second logistic regression model computed based on the coefficient (B) statistics.

^c^The normalised version of the statistical weight is introduced in the previous column.

Table 1 shows the combined results of the individual models. These results represent the average precision of each model and their absolute and normalised statistical weights. After computing SI based on the presented models’ results, the overall performance of the pipeline achieved a SI of 0.81. The closer the SI value to 1, the more accurate the framework is in detecting the different risk factors in accordance with the different precision of the deep models and the weight of a given factor on the occurrence of near misses. Based on the results of the normalised weights, it is worth mentioning that the SI index is highly influenced by the precision of scenes that belong to clear and rainy weather and those that include people and bikes. Nevertheless, it is less influenced by the precision of scenes that include trucks, glare, and wet surfaces.

### Association between variables

**Fig. 2:**
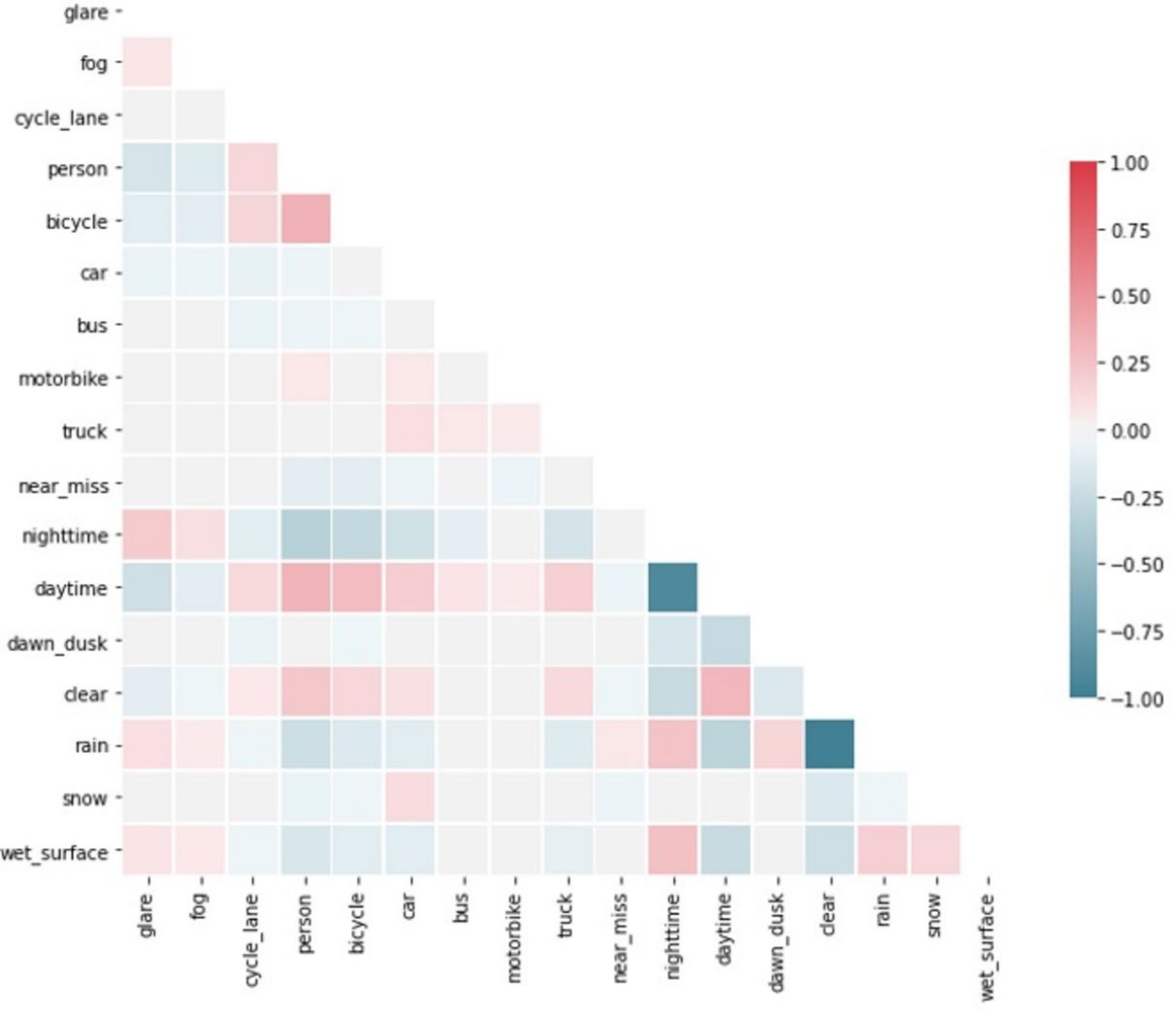
Correlation matrix of key variables using the product moment correlation coefficient (PMCC). This figure is made by the Author, Mohamed R Ibrahim.

We have used the Product Moment Correlation Coefficient (PMCC) to highlight the linear correlation in the data set. The PMCC measures the correlation between two variables in the range $[-1,1]$, where 1 represents a perfect positive correlation, $-1$ represents a perfect negative correlation and 0 represents no correlation. For further explanation of the PMCC, see^23^. Figure 2 shows the PMCC between each pair of variables. It indicates a different positive and negative correlation, in which some of them can be considered as new findings, whereas others can be seen as logical and expected outcomes. For instance, daytime is inversely correlated with night-time, and clear weather is inversely correlated with rainy weather, which is logical and expected. Similarly, the presence of people is positively correlated with daytime, clear weather, and the presence of bicycles. The presence of glare is positively correlated with night-time, rain, and fog. While glare is usually associated with sunny conditions, the detected glare in this dataset is due to headlights in darker conditions such as rain and fog. Wet ground is positively correlated with rain, snow, and night-time. On the other hand, a crucial finding is that near misses are positively correlated with rain but uncorrelated with daytime. Furthermore, while there is a positive correlation between the presence of a cycling lane and the presence of people and bicycles, there is an absence of correlation between the presence of a cycling lane and the occurrence of near misses. It may seem counter-intuitive that dawn/dusk and daytime are not perfectly negatively correlated, so it is worth mentioning that this is because there are three mutually exclusive classes (Dawn/dusk-time, daytime, and night-time).

As a step forward to further investigate the collinearity in the data set among the different variables, we used a t-test to highlight the significant differences between the near miss and safe scenes in terms of the selected variable. The t-test method is used to compare the means of the continuous variables for both groups, safe and near misses. When the p-value is less than 0.05, the null hypothesis can be rejected, and the results of selected variables can be deemed statistically significant. It can be used to differentiate between safe and near miss scenes. For further explanation regarding t-test analysis, see^24,25^. Table 2 shows the statistically significant results of the t-test method for five significant independent variables, in which the near miss variable is treated as a dependent variable. The results show that the occurrence of near misses is statistically significant with the counts of cars, buses, and motorbikes with positive coefficient values and statistical significance with the counts of people and bicycles with negative coefficient values.

**Table 2:** The significant results of the t-test method.

Variable	F-statistics	P-value
Car	15.497	0.000
Person	− 32.137	0.000
Bus	12.192	0.000
Bicycle	− 33.540	0.000
Motorbike	2.529	0.011

### The impacts of risk factors in cycling near misses

We aim to directly grasp the influence of various independent variables on the occurrence of near misses in non-controlled experiments. This stage serves as a foundational model for subsequent sections where we delve deeper into specific variables that have demonstrated a statistically significant relationship with near misses.

There are 13,145 frames categorized as near misses and 33,422 frames identified as safe cases. To achieve a more balanced representation of the dependent variable, we initiated a new experiment to address the class imbalance. This involved drawing a random sample of 13,145 from the 33,422 safe case frames. Using a Logistic regression model, we assessed the collinearity between the near miss variable (dependent) and other independent variables, without considering any confounder assumptions or controlled variables. For a comprehensive understanding of logistic models and utility functions, refer to^26^ and^27^. Table 3 summarises the statistics of the Logistic regression model. There are several independent variables are identified as statistically significant, with various variable coefficients and standard errors. This model shows a statistically insignificant intercept. The table shows the coefficients of all variables and their statistical significance with the occurrence of near misses in presence of a cycle lane.

**Table 3:** The results of the logistic regression model (balanced classes of near misses).

$\hbox {Variables}$^a^	Coefficient (B)	Std. error	Wald	Pvalue	Exp(B)	Lower	Upper
Intercept	0.164	0.129	1.613	0.204			
Person	0.074	0.006	139.597	0.000	1.076	1.063	1.090
Bicycle	0.201	0.016	153.051	0.000	1.223	1.184	1.262
Car	0.089	0.010	73.561	0.000	1.093	1.071	1.115
Bus	− 0.100	0.029	11.928	0.001	0.905	0.855	0.958
Motorbike	0.368	0.054	46.338	0.000	1.445	1.300	1.607
Truck	0.034	0.031	1.196	0.274	1.035	0.973	1.100
Dawn/dusk-time	− 0.417	0.063	43.899	0.000	0.659	0.583	0.746
Day-time	− 0.120	0.032	14.394	0.000	0.887	0.834	0.944
Night-time	0^b^						
Glare = 0	− 0.099	0.034	8.418	0.004	0.906	0.848	0.968
Glare = 1	0^b^						
Weather = clear	− 0.117	0.098	1.433	0.231	0.889	0.734	1.078
Weather = rainy	− 0.281	0.099	7.994	0.005	0.755	0.622	0.917
Weather = snowy	0^b^						
Fog = 0	− 0.241	0.073	10.812	0.001	0.786	0.681	0.907
Fog = 1	0b						
Cycle_lane = 0	− 0.044	0.026	2.790	0.095	0.957	0.909	1.008
Cycle_lane = 1	0^b^						
Wet_surface = 0	0.027	0.052	0.275	0.600	1.028	0.928	1.138
Wet_surface = 1	0^b^	0.129	1.613				

^a^The reference category for the independent variable (near misses) is 1.

^b^This parameter is not included in the model. Model parameters are: $N= 26290$ and $R^2 = 0.027$.

### Granger causality of risk factors

**Fig. 3:**
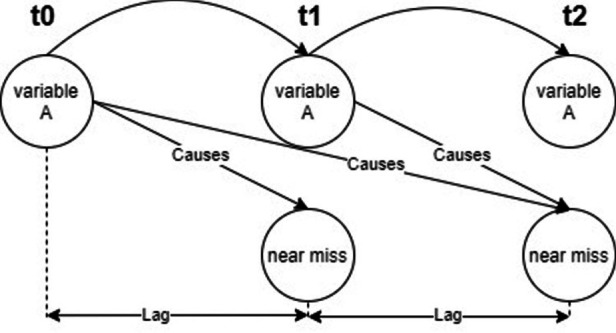
The assumption for Granger Causality. This figure is drawn by the Author, Mohamed R Ibrahim.

Understanding the temporal causal structure of a given dataset is essential for interventions and decision-making for real-world applications^28^. For a given risk factor to cause a near miss, it has to precede its occurrence. If the time lag between the risk factor being observed and the near miss occurring can be modelled, then it has the potential to be used in an early warning system. Figure 3 shows the assumption of temporal causality, highlighting the scope that defines causality. To test for granger-causality, the figure shows that the tested variable must be in a sequential form and there is a defined lag between the selected variable and a near miss for causality to be significant. To compute Granger causality for the different independent variables (16 variables), different experiments have been conducted for selecting a lag value. We experimented with values in the range of 1 to 120. This selection is made based on (1) trial and error and (2) the nature of the data set used, in which 30 data points represent 1 s. The results show statistically significant outcomes for three independent variables (car, person, and glare), which means that the count of cars, persons or the occurrence of glare Granger-causes near misses for different lag intervals. In other words, these variables could be useful for forecasting the occurrence of cycling near misses.

Table 4 shows the result of the Granger causality for these three variables. Firstly, regarding the car variable, the results show significant Chi-squared and F-test values at a p-value less than 0.05 for a lag value that is 17 or lower (below 0.5 s). Besides the significant causality, this could also indicate the short-term effect or the rapid effect of the presence of a car in Granger-causing the occurrence of near misses. Secondly, regarding the person variable, similar to the car variable, the results show a statistically significant chi-squared test and f-test for various lags of p-value below 0.05. However, unlike the car variable, the causal effect of the person variable has a long-term effect in which the lag values range from 18 to 42 (approx. 1.5 s). Lastly, regarding the glare variable, similar to the two aforementioned variables, the occurrence of glare shows a statistically significant chi-squared test and f-test at different lags. Unlike these two variables, however, the causal effect of glare on the occurrence of near misses remains significant in both the short-term (0.5 s) and long-term (2 s). This could indicate how crucial the existence of glare is to the occurrence of cycling near misses.

**Table 4:** The significant results of the Granger causality models.

Variable	Lag	F-test	Chi-squared test	P-value
Car	5	2.5048	12.5269	0.0283
	7	2.4416	17.0964	0.0168
	8	2.2060	17.6547	0.0240
	9	1.9930	17.9443	0.0359
	14	1.8116	25.3778	0.0312
	15	1.6962	25.4597	0.0443
	16	1.7422	27.8947	0.0328
	17	1.6529	28.1212	0.0438
Person	18	1.6205	29.1928	0.0464
	25	2.1248	53.1480	0.0009
	42	1.4345	60.3207	0.0337
Glare	26	1.5611	41.0922	0.0342
	60	1.4308	85.9895	0.0160

## Discussion

This research introduces an overall methodology of different deep and mathematical models in an integrated pipeline. The general goals of this framework are to detect critical issues in cities, such as cycling near misses while extracting their risk factors and their effect on these critical events. It introduces a framework stringency index that aims to evaluate the overall methodology, in addition to the evaluation metrics conducted on the individual methods and models. The importance of this index can be highlighted in evaluating the weights and the importance of the individual models in their function and utility in the overall methodology. Nevertheless, the number of outputs that each sub-method contributes to the overall methodology. Last, the research also highlights the importance of the flexibility of the introduced pipeline that could allow and cope with any future adaptation, either in terms of refining methods, or introducing new ones. After analysing a generated dataset of a large sample (N= 46,567), we analysed the impacts of several risk factors on cycling near misses, which we focus here on linking these findings to the literature, highlighting future work.

### Linking results to literature

When it comes to the physical state of the built environment, it has been observed that in the presence of a cycle lane, drivers may travel inside their designated lane with less regard for ensuring a safe passing distance for cyclists in the adjacent cycle lane^29^. A recent investigation on close pass events corroborated similar outcomes^30^. However, we found that, based on the statistical significance of the regression model, near misses are less likely to occur in the presence of a cycle lane in comparison to its absence. When we examined this, we discovered that both weather and surface conditions had statistical relevance. These findings are in line with the literature. For example, several studies include surface condition factors such as wet, dry, well-maintained, or deteriorating surfaces^1,31–34^. It was discovered that some near misses occurred at the icy surface^35^. It was also revealed that the majority of the close misses occurred on dry surfaces^33^. These frequencies, however, are based on the count of responses rather than the relevance of the finding, which could be related to the self-selection of travel routes or duration. In our study, we looked at how often road users are involved in near misses, finding their statistical significance. These findings fill another gap in the literature by directly investigating the impact of the surrounding context on the flow of traffic for bicycles, pedestrians, and vehicles in a specific area in cities, potentially exposing cyclists to risk^2,36^. It has been demonstrated that a lack of exposure data makes it difficult to draw meaningful conclusions^2,36^. They also stated that because this data frequently overlooks minor accidents, near miss events are more likely to be missed as well, making it impossible to assess safety standards between different types of infrastructure. Last, even though the time of day may be a substantial risk factor in cycling accidents^2,37^, many studies completely ignore the problem of time^1,11, 17, 38–44^. Other studies categorise visual conditions as either day or night, without taking into account more complex effects as those brought on by direct sunlight (i.e., glare) at dawn and dusk, which our findings aimed to contribute to this knowledge gap.

### Real-world applications

This study’s innovative approach to analysing cycling near misses has several far-reaching applications that could transform urban cycling safety. Primarily, by providing real-time analysis, the framework enables immediate responses to risky situations, which can help city planners and traffic authorities to swiftly implement safety measures and adjust traffic regulations as needed in a given location. This proactive approach can significantly reduce the incidence of cycling-related accidents and support the expansion of cycling as a commuting mode. Moreover, the detailed insights gained from the study allow for more informed decisions in urban planning. By identifying specific risk factors and risky areas, planners can design cycling paths and urban layouts that minimise these risks, potentially including features like protected bike lanes and improved lighting at critical intersections. In addition to infrastructure planning, the framework supports the creation of dynamic and informative awareness campaigns. These campaigns can use data from the study to highlight specific behaviours that lead to near misses and advocate for best practices among cyclists and motorists alike, such as the use of proper signalling and the importance of maintaining a safe following distance. Another key application of this study is the development of advanced risk mapping. Such maps not only show safer routes for cyclists but also integrate real-time data to update risk levels based on factors like time of day, weather conditions, and traffic volume. This can empower cyclists with the information needed to make safer travel decisions. Finally, the framework developed in this study could also serve as a model for other modes of transportation (i.e. motorcycles), where similar near-miss analysis frameworks could be implemented to enhance overall traffic safety and efficiency. By extending these methodologies beyond cycling, the potential benefits of this research could contribute broadly to smarter, safer urban mobility solutions.

### Limitations

This research presented new approaches and outcomes for understanding the contributions of risk factors such as the counts of road users, visual, weather or surface conditions to the occurrence of cycling near misses. However, there are still limitations that need to be addressed in future work when it comes to assessing the cause and effect of the stated subject. First, data representation and distribution: Finding observation points that represent various types of events and conditions in the scope of the stated subject remains a critical issue for understanding and generalising the measured causes and effects. In this vein, for future studies, a naturalistic study needs to be carried out to include a representative sample of data that belongs to different types of near misses, and different visual, weather, and physical conditions. Second, addressing the behavioural represents another limitation. Similar to addressing the issue of representative data in terms of scene types and conditions, the representation of strata that belongs to different socioeconomic structures needs to be considered.

## Methods and materials

For cycling near misses video streams, we utilised a subset from the dataset provided by^22^. The dataset contains 46,567 sequential frames, representing 209 unique near miss videos with an average duration of 1.3 s. The dataset includes a refined selection of video clips that capture a broad range of environmental, temporal, and visual contexts for urban cycling scenarios. This study seeks to expand upon previous research by exploring the dynamics and risk factors associated with cycling near misses, going beyond mere detection of these events as outlined in the previous study^22^. The dataset focuses on the characteristics of the near misses, such as timing, environmental conditions, and interactions with various road users. By using sophisticated statistical tools and machine learning algorithms, the research identifies patterns and trends that could inform safer urban planning and cycling infrastructure. Moreover, the paper evaluates the effectiveness of existing models in detecting and classifying different types of near-miss events and suggests improvements based on the insights gained from the secondary dataset.

### Proposed framework

**Fig. 4:**
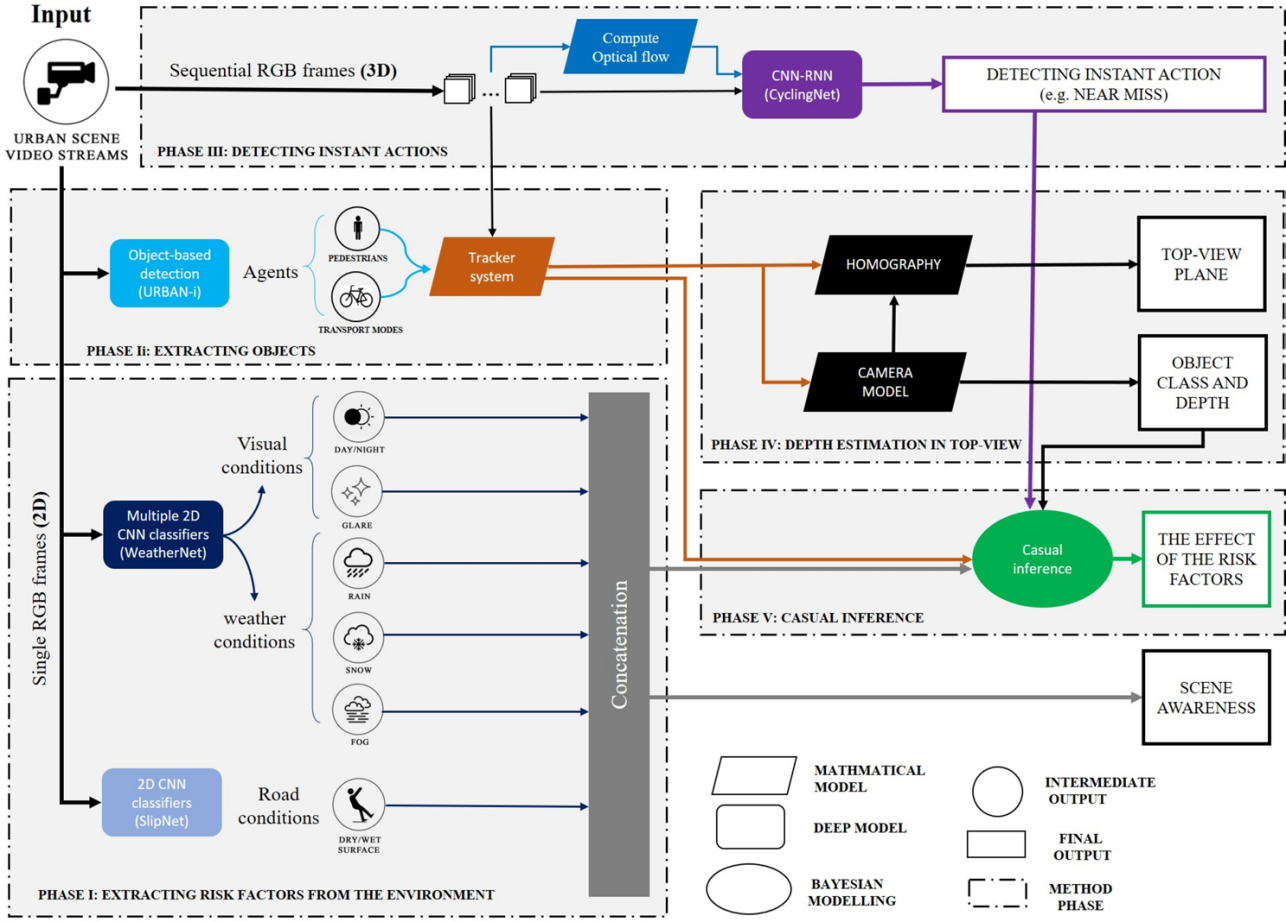
The introduced pipeline for analysing cycling near misses from video streams. This figure is drawn by the Author, Mohamed R Ibrahim.

There are different approaches for integrating different tasks which depend on the availability of multi-label data, the ability of fusing data of different input parameters, or the availability of computational resources. Multitask and ensemble learning are two crucial approaches for learning multiple tasks^45^. Multitask learning refers to the simultaneous training of several tasks of the same input, in which tasks can share intermediate-level representation in some shared layers. This approach aims to improve generalisation by pooling the examples outputted by several tasks^45^. On the other hand, ensemble learning refers to combining multiple models to solve a given problem. There are different purposes of ensemble learning, most commonly, the bootstrap aggregating (or bagging) technique^45^. In this approach, several models are trained differently for a given task and combined to reduce generalisation errors. Ensemble learning, however, is also used for other purposes such as data fusion or incremental learning^46^. Certain problems can be too difficult for a given classifier to solve or too computationally expensive to conduct, in which case the divide-and-conquer approach can be utilised through incremental learning. Accordingly, ensemble learning seems suited to the diversity of computational tasks required to recognise cycling near misses and their risk factors. Different tasks can be learned by the representation of the input incrementally. This approach will allow flexibility in how the input data can be used and organised for each given task and minimise the computational requirements of training several models for various tasks at once. It would also allow modification and further development, at a later stage, of any given model without affecting the other assembled tasks.

The introduced framework is built based on ensemble learning with a single input of video streams. The framework outputs four outcomes: (1) critical event detection (in this case, near misses), (2) a list of detected risk factors and objects, and last (3) causal inference for the detected factors on the detected critical event. The pipeline is fully coded in Python programming. After training, testing, and validation, the pre-trained deep learning models are utilised for analysing future scenes as a pragmatic computer vision tool. For the objectives of this research, there are multiple advantages to selecting ensemble learning. During the training phase, this approach allows various tasks to be trained separately based on their input and computational requirements or the availability of data that might not be possible with other approaches such as Multitask learning. At inference, it allows the single input to be treated differently throughout the pipeline as either single-frame images or sequential images based on the specific tasks. In the post-production phase, ensemble learning allows the pipeline of the framework to be modified or expanded for a given task without affecting the other models in the pipeline.

Figure 4 shows the overall workflow of the proposed pipeline when a video stream is received as input. First, phases I and II extract risk factors and agents (pedestrians, cycles, vehicles etc.), respectively, while phase III detects instant actions (near misses) in parallel. The outputs of all preceding phases are then fed into phase IV, where causal inference is performed. The four phases are described in detail in the following subsections:

#### Phase I: Extracting risk factors from the environment

This phase tackles the different factors related to the environment that may influence the safety of the cyclist. Alongside image classification, understanding the overall gist of a scene is crucial for understanding the built environment^47^ and few studies have been done in this area. For instance, sensing the qualitative measures that are related to the built environment that may contribute to near misses, such as road infrastructure, lighting and weather conditions. Weather and visual conditions are often addressed individually. WeatherNet^48^ introduces a novel framework to automatically extract this information from street-level images relying on deep learning and computer vision using a unified method without any pre-defined constraints in the processed images (i.e., pre-determined field of view, angle, positioning, or cropping). The WeatherNet model comprises four ResnNet models to extract various weather and visual conditions such as Dawn/dusk, day and night for the time of day; glare for lighting conditions; and clear, rainy, snowy, and foggy for weather conditions. Moreover, wet road conditions, combined with other factors related to visibility, weather and/or physical conditions may contribute to many risky situations and instant events when it comes to mobility in a complex environment. Whether driving, cycling, or even walking, a wet surface may cause potential near misses, or serious incidents. The classification of the road is often interpreted based on the perceived weather and precipitation conditions. However there may be cases where the ground is wet enough to cause a critical event while the sun is shining, and conversely, there may be rainy days where the ground is not yet wet. To tackle this subtle issue, we trained a model similar to WeatherNet to detect the whether a given road is wet or dry. Additionally, we trained a model to detect the presence of a cycling lane. We followed the same training implementations suggested by^48^.

#### Phase II: Detecting and tracking objects

We introduce simultaneous object detection and tracking of road users to the overall framework. The phase consists of two main models: (1) Object detection and (2) multi-object tracking. To detect road users (i.e. people, cars, trucks, buses, motorcycles, and bicycles) from extracted scenes, we used You Only Look Once (YOLO) V5 method^49^, trained on COCO dataset^50^. After object detection, we utilisied Deep Simple Online and Realtime Tracking (DeepSORT) method^51^. The SORT method is suitable for online object-tracking because (1) Its speed allows fast computation without a huge drop in Frames Per Second (FPS), (2) it relies on simple techniques such as Kalman Filter, which makes it easy to implement online without previous training. In the case of tacking, the SORT method is evaluated on Multi-Object Tracking (MOT) benchmark datasets^52^.

#### Phase III: Detecting instant actions

In this phase, we utilise a new method called CyclingNet^22^ for detecting cycling near misses from video streams generated by a mounted frontal camera on a bike. CyclingNet is a deep computer vision model based on a convolutional structure embedded with Self-attention Bidirectional Long-short Term Memory (LSTM) blocks that aim to understand near misses from both sequential images of scenes and their optical flows. The model is trained on scenes of both safe rides and near misses. Action recognition, relying on the CyclingNet model. For further details of how the model is developed and trained, see^22^.

#### Phase IV: Causal inference

We aim, after precisely extracting a combination of risk factors, to understand: (1) the cause and effect of individual risk factors on the detected events, (2) the causality of these risk factors in the detected events in a time series. Accordingly, this phase relies on statistical modelling techniques to uncover the causes and the effects of each extracted factor on the detected events. It includes two types of analysis, which will be covered.

##### Logistic regression

 We use the detected variable corresponding to critical events as the dependent variable, with detected objects and risk factors being independent or control variables in a logistic regression model. The utility function of the near miss category $i$ in the occurrence of $j$ is computed as:

1 $$\begin{aligned} v_{ij} = \varepsilon + \sum _{k \in T} b_k x_{ijk} \end{aligned}$$

where $x_{ijk}$ represents the attribute $k$ for point $j$ on near miss occurrence of $i$, $b_k$ is a coefficient in the utility function, $T$ represents the set of attributes, $\varepsilon$ represents the stochastic part of the utility function.

The coefficient of the model is computed by estimating the maximum likelihood, whereas the stochastic part $\varepsilon$ is computed by assuming it as a double exponential distribution. The logarithm of the likelihood of the model of the actual occurrence of near misses can be expressed as:

2 $$\begin{aligned} \log L = \sum _{i=1}^{N} \sum _{j=1}^{J} Y_{ij} \ln P_i(Y=j|x,\beta ) \end{aligned}$$

where:

3 $$\begin{aligned} P_i(Y=j|x,\beta ) = \frac{\exp (v_{ij}(x_{ij,b}))}{\sum _{h=1}^{j} \exp (v_{ih}(x_{ih,b}))} \end{aligned}$$

where $Y$ is the binary dependent variable, $X$ represents the independent variables, $v_{ij}$ is the utility function for the $j$-th alternative of $i$-th choice, $N$ represents the occasion of choices, $j$ represents the number of alternatives, $P_i$ represents the predicted probability of the occasion of $i$ occurrence of a near miss, and $\beta$ represents the parameter vector of the model.

We also include different parametric and non-parametric tests (i.e. t-test) to determine the strength and significance of the results.

##### Granger causality

 Granger causality is a probabilistic method for investigating the causality between two variables in a time series dataset. Unlike understanding the general cause and effect of the individual factors, causality, or the ‘Granger-cause’, focuses on highlighting when a particular variable comes before another in time series data. The Granger causality method is employed^28,53^. Granger causality is a statistical approach used to determine whether a given time series could be useful in predicting another one. The main hypothesis is that if a time-series $X_1$ Granger-causes a time-series $X_2$, then the past values of $X_1$ should contain information that assists in predicting $X_2$. To avoid the post hoc fallacy (Given that an event x is followed by an event y, event x must have caused event y), Granger causality aims only to find predictive causality, whereas true causality is rather a philosophical argument. Given that the variables are extracted from sequential frames, each variable can be seen as a time series and this approach can be useful to determine whether any risk factor Granger-causes near misses based on the time lag between the occurrence of a near miss, and the preceding existence of a given risk factor. To compute Granger causality, the variables have been transformed to stationary series, ensuring that the data distribution (mean, variance, and autocorrelation) of the variables do not change over time.

##### Framework stringency index

 The performance of each model introduced in the pipeline of the overall methodology is evaluated with different metrics and loss functions depending on the types and scopes of the given task of the model. Nevertheless, the models not only vary based on their evaluation metrics but also the resulting accuracies and precisions. On the other hand, as shown in Fig. 4, The relations between these different models vary. For instance, some models function consecutively, while others function in parallel to other phases. The goal of this research is to provide a stable framework to be used as a computer vision tool for the detection of near misses, risk factors, and their effects on near misses. This makes it a challenge for a pipeline of mixed models and different ensemble techniques to be evaluated as a whole. Traditionally, the performance can be measured based on the sum of the losses of each model, when models are evaluated similarly, and hold the same weights of utilisation in the entire pipeline. Given that we aim to develop a verified pipeline of the different pre-trained models, we introduce a new stringency index to indicate the performance of the entire framework on a given input that can draw a conclusion based on three aspects: (1) the individual loss of each model, (2) the number of outputs of each model, and 3) the weight of the utilisation of each model in the framework. The framework Stringency Index (SI) is defined as:

4 $$\begin{aligned} SI = \sum _{i=1}^{t} \sum _{o=1}^{n} \sum _{i=1}^{j} \left( \frac{\beta _i \cdot P_i}{t} \right) \end{aligned}$$

where $j$ denotes the number of outputs per model, $n$ denotes the number of models in the framework, $t$ represents the total number of sequential frames, $P$ represents the estimated average precision between the predicted and actual value for a single output $o$ of a given model $i$, and $\beta$ represents the normalised statistical weight of a given risk factor on a given task of the detection of a near miss.

## Remarks

The general goals of the introduced framework are to detect critical issues in cities, such as cycling near misses, while extracting their risk factors and their effect on these critical events. This paper introduces a framework stringency index that aims to evaluate the overall methodology, in addition to the evaluation metrics conducted on the individual methods and models. The importance of this index can be highlighted in evaluating the weights and the importance of the individual models in their function and utility in the overall methodology. Nevertheless, the number of outputs that each sub-method contributes to the overall methodology. Last, the paper also highlights the importance of the flexibility of the introduced pipeline that could allow and cope with any future adaptation, either in terms of refining methods, or introducing new ones.

## Data Availability

The raw video dataset is provided by^22^, all processed data is available upon request from the corresponding author on reasonable request.
